# Streamlining Hospital Planning: A User‐Friendly Decision Support Tool for Enhanced Healthcare Delivery

**DOI:** 10.1002/hpm.70120

**Published:** 2026-07-27

**Authors:** Eren Demir

**Affiliations:** ^1^ Hertfordshire Business School University of Hertfordshire Hatfield UK

**Keywords:** decision support tool, discrete event simulation, hospital capacity, hospital planning

## Abstract

Healthcare providers worldwide face challenges in maintaining a delicate balance between rising demand and limited resources. Decision‐makers often struggle with planning and implementing new services, leading to costly consequences and hindering efficiency, patient outcomes, and staff well‐being. Current systems lack a comprehensive modelling approach that encompasses all aspects of the hospital and fails to generate crucial outputs. Moreover, these tools often require expert modelers, limiting their usability for end users such as service managers, clinicians, and management. To address this gap, a user‐friendly decision support tool (DST) is developed that empowers key decision‐makers to assess the impact of interventions across all hospital operations. Our DST utilises a discrete event simulation model, encompassing specialties and interconnections among hospital services. The model incorporates a dataset of 360 input parameters and statistical distributions, providing a holistic understanding of operations. Through scenarios from 2023–2027, our DST generated diverse outputs, including activity levels, bed and clinic requirements, theatre utilization, and financial implications. Notably, implementing a virtual ward reduced patient backlogs and eased strain on resources from 113% to 80%. Our adaptable DST enables informed decision‐making, enhancing patient outcomes and optimising resource utilization. This user‐friendly tool revitalises hospital operations for improved efficiency and effectiveness.

## Highlights

- Tackles acute healthcare challenges due to increased demand.
- Offers insights into healthcare key performance indicators, aiding evidence‐based decision‐making.
- Captures the interconnected nature of an entire hospital system.
- Recommends virtual wards to optimise elective care and outpatient services.

## 1 Introduction

The global healthcare sector has faced numerous challenges because of an uneven distribution of healthcare service availability and demand. These problems have highlighted the importance of establishing effective solutions that can ensure the prompt and efficient provision of healthcare services to cater to the diverse needs of patients. To ensure the provision of high‐quality healthcare to patients, healthcare providers must identify and address the underlying causes of these problems [1]. Healthcare delivery in the United Kingdom is currently facing a number of workforce‐related challenges [2], including increased staff absences due to illness and early retirements, some of which are a result of pension issues for doctors [3], as well as the effects of Brexit and reduced immigration due to the pandemic [4]. The UK currently has a shortage of roughly 40,000 nurses, which is predicted to rise to over 70,000 by 2023 [5].

The shortage of healthcare workers is not unique to the UK. In the US, for example, there is a projected shortage of up to 500,000 nurses by 2030, as well as a shortage of clinicians and other healthcare professionals [6].

Furthermore, the healthcare systems of European nations are under increasing strain as a result of an aging population and an increase in chronic disease cases [7]. This growing burden exacerbates the issues that already overburdened healthcare systems are facing. Demand for healthcare services has exacerbated the problem, as indicated by a large increase in patient demand for healthcare services. Following a period of reduced activity during the pandemic, there seems to be a rebound effect in demand for hospital services in the UK [8]. Hospital emergency room attendance is at record levels, and as of January 2023, in England, the number of patients awaiting treatment reached 7.2 million, marking a significant increase from the pre‐pandemic figures in December 2019, when the waiting list exceeded 4.5 million, reflecting a 60% surge [8]. The situation in emergency departments is beyond critical, and as of December 2022, there were 54,532 patients waiting 12 hours or more for treatment in England, representing a significant increase of 60 times compared to the combined total from 2011 to 2014 [8].

To address the challenges facing healthcare systems around the world, various solutions are being proposed, such as integrated care models (ICM) to enhance coordination among healthcare providers to streamline care delivery [9] and effective workforce planning and resource management to ensure sufficient staffing levels are available for optimal care delivery [10].

A systematic review conducted by Mbau et al. [11] identified numerous global solutions to enhance health system efficiency. Most studies originated from high and upper‐middle‐income countries, benefiting from robust data availability. Key solutions, such as ICM and workforce strategies, were highlighted (see Table 1 for details). The review underscores the importance of contextualizing policy options to specific settings. It highlights the need for a whole‐system view (i.e., ICM), to ensure that reforms are effective within their policy environments.

**TABLE 1:** Solutions for enhancing health system efficiency as identified by Mbau et al. [11].

Key solution	Description
Multisectoral coordination	Strengthening coordination across sectors is essential, as health systems are influenced by broader demographic, socioeconomic, and political factors. This calls for integrated approaches to health that go beyond the health sector alone.
Preventive and promotive health interventions	Investing in preventive and promotive health measures is cost‐effective and reduces the burden on health systems. Health policy reforms should prioritise these interventions alongside optimising health system functions.
Health governance and financing reforms	Strengthening health sector leadership, decentralising health functions, and regulating healthcare costs are vital for improving efficiency. Financing reforms such as scaling up prepayment mechanisms, defining evidence‐based benefit packages, and reforming payment systems are also recommended.
Optimising health system inputs	Ensuring an adequate and equitable distribution of health workers, commodities, and infrastructure is key. Policy options include reorienting health systems towards primary health care, strengthening community health systems, and improving gatekeeping and referral systems.
Quality of care and avoiding overprovision	Interventions to enhance care quality and reduce unnecessary services are necessary. This includes avoiding inappropriate hospital admissions, medical errors, and overuse of procedures and equipment.

ICM is emerging as a promising field, emphasising the seamless integration of healthcare services to elevate patient care while addressing fragmentation [12]. This approach offers a holistic perspective on the patient journey within healthcare systems, aiming to facilitate smoother experiences across diagnosis, treatment, care, rehabilitation, and overall health outcomes [13]. By prioritising localised delivery models tailored to meet specific population needs, integrated care has the potential to unite diverse sectors, spanning health, social care, mental health, primary care, and community services, thereby fostering a comprehensive approach to healthcare delivery [14].

Existing literature also indicate that ICM alone is not a sufficient solution without a well‐developed workforce and resource management plan [11]. Healthcare workforce planning is a critical aspect of health system management. According to the World Health Organization's guidelines [15], robust workforce planning should aim to ensure that the right number of healthcare professionals with the appropriate skills are available to meet the needs of the population. Existing healthcare workforce modelling methods primarily include mathematical methods and computer simulation methods. These methods serve different purposes in coping with the complexities of workforce planning [16]. For example, an integer programming model was developed to cope with medical staff scheduling problems for the emergency medical service system by minimising the total cost and workforce supply [17].

According to Park [18], optimising workforce planning requires considering economic efficiency, balancing costs with patient outcomes, such as mortality and quality of care, and ensuring an appropriate mix of staff, including registered nurses and assistant nurses. An optimal nurse staffing model was developed using linear programming to optimise the workforce in terms of quality of care, nurse staffing levels, and costs [19].

Similarly, an agent‐based simulation model was developed to determine the nurse staffing levels in NHS hospitals in England [20]. In addition, Abas et al. [21] developed a system dynamics model to predict the future workforce supply. The workforce life cycle flow was analysed with interactions between the training model, the population model, and the full‐time equivalent staff model.

Despite extensive research conducted on potential solutions, to date, there has been a dearth of research examining the effects of these solutions on healthcare service delivery in greater detail. Hospitals provide a wide range of services, and it is unclear how implementing new solutions would affect critical resources like bed capacity, operating theatre utilization, staffing requirements, and financial implications. The complexity of healthcare systems means that modifying a single element does not always result in an overall improvement. The literature suggests that adopting a whole‐system view is essential due to the interconnected nature of multiple factors that need consideration. For instance, emergency departments are linked to all other services and specialties, and an increase in emergency attendance could result in appointment cancellations for planned admissions. Therefore, any changes made to emergency departments must consider all planned and unplanned admissions, including outpatient attendances. It is crucial to evaluate potential changes before implementation to provide local healthcare services, managers, and staff with empirical evidence that supports the notion that implementing innovative strategies, interventions, and policies will result in desired outcomes. This allows the changes to be implemented with confidence.

This article introduces a user‐friendly decision support tool (DST) designed to facilitate hospital planning and empower key decision‐makers to evaluate the effects of changes prior to their implementation. The DST is capable of generating a range of outputs, including patient outcomes (e.g., readmissions, disease progression, quality‐adjusted life years [QALYs]) and resource utilization metrics (e.g., bed requirements, clinic utilisation, and financial implications). Both sets of outputs are equally significant, providing a comprehensive understanding of operational efficiency and patient care quality within the hospital. While the primary focus of this article has been on operational metrics to support key decision‐makers in streamlining hospital planning processes, it is important to acknowledge that the simulation also robustly generates patient outcome metrics.

This article outlines the study's background, objectives, methodology, data sources, and results. The study focuses on alleviating pressures experienced by an NHS service provider in England, but the DST is adaptable and can be applied to hospital services globally.

## 2 Background and Objectives

Due to the inherent complexity of hospitals, efficient hospital management is critical. To do this, the entire system, including patient interactions and resource utilization at each stage of care across inpatient, outpatient, and emergency care, should be carefully modelled. Tracking the resources consumed at each stage of the patient flow, such as waiting times for appointments, the time spent by doctors and nurses, the use of diagnostic procedures, and the interactions between services and specialties, will facilitate a more profound comprehension of the hospital system and uncover potential areas for improvement.

In the healthcare sector, simulation techniques have been frequently utilised to evaluate the possible magnitude of improvements that can be achieved through the implementation of new strategies and interventions. These approaches, which include agent‐based simulation (ABS), discrete event simulation (DES), and system dynamics (SD), have proven to be more effective than analytical solutions such as optimization [22], Markov modelling [23], and queuing theory [24] in modelling complex healthcare systems. Analytical‐based models are developed to solve a well‐defined mathematical problem, which can be very restrictive in terms of capturing complex systems like a hospital, whereas simulation models are generally characterised by their flexibility [25].

DES is appropriate for simulating operational procedures that contain resource restrictions and uncertainties, and it enables the detailed implementation of individual patient movement inside a hospital system [26]. On the other hand, SD is a method used to examine complex healthcare systems. Instead of diving into the day‐to‐day operations, it zooms out to analyse the bigger picture and strategic aspects of the system [27]. It helps us understand how various factors interact and influence the overall functioning of the healthcare system. ABS focuses on studying how individuals (such as patients) behave within a healthcare system and how their interactions can influence the overall functioning of the system. It examines how changes in individual behaviour can have an impact on the system as a whole [28].

DES is usually chosen as the simulation methodology because of its ability to capture uncertainty, the resource‐intensive nature of the hospital care system and generate operational related key performance metrics for day‐to‐day decision making. It simulates the patient's flow and processes at discrete moments in time, making it suitable to be applied and implemented in practice in various health and social care settings.

DES is widely embraced due to its ability to accurately represent care pathways and track the sequential movement of patients within these pathways. In the model, patients are treated as distinct entities, and their progression through various stages of the pathway is continuously monitored as time passes [29]. Moreover, DES provides a means of representing uncertainties that are typical in healthcare delivery, such as the likelihood of patients being readmitted after being discharged. A DES model can also incorporate individual characteristics such as sex, age, severity, length of stay, and time to treatment, which can influence both the progress of patients along the care pathway and the ultimate results of their treatment [30].

Furthermore, DES allows the testing of what‐if scenarios to examine alternative scenarios that represent new policies and interventions, such as the implementation of virtual wards. Utilising the DES model enables the simulation of these scenarios, enabling the prediction of potential performance enhancements at an operational level when implementing strategies, interventions, and policies. This empowers decision‐makers with valuable insights and evidence to guide their implementation of reforms and enhance the decision‐making process.

The literature is replete with DES models addressing specific challenges in healthcare. Based on recent comprehensive literature reviews conducted separately by Vazquez‐Serrano et al. [31] and Forbus & Berlant [32], DES studies can be categorised into three areas: healthcare systems operations (HCSO), disease progression modelling (DPM), and screening modelling (SM) [32]. HCSO is concerned with demand and capacity modelling of services, such as *resource allocation*, of which hospital bed capacity planning is the most popular, the effects of *operational change, and patient and staff scheduling*. DPM focuses on the course of a disease with particular attention on economic modelling, evaluating the effectiveness and cost‐effectiveness of a treatment (typically using quality‐adjusted life years as a key metric of interest) so that well‐informed decisions can be made in the allocation of treatment resources prior to investing time and money. Finally, SM is conducted to analyse the impacts of modifying the methods used to identify individuals with a specific disease or the determination of whether they qualify for a specific care pathway, such as screening for HIV, cancer, and tuberculosis.

The studies suggest that a comprehensive model incorporating the day‐to‐day operations of a complete hospital, including emergency, outpatient, and inpatient services, is exceptionally uncommon. To date, there is a scarcity of decision support tools (DST) that provide a comprehensive representation of an entire hospital within the context of healthcare. Gunal and Pidd [33] have developed a notable simulation model that captures performance‐related outputs, specifically focussing on referral‐to‐treatment consultant‐led waiting times, which is an essential metric for evaluation. However, senior decision‐makers in healthcare require a broader range of metrics to effectively plan and allocate resources. These metrics include human and non‐human resource requirements, such as bed, clinic, and theatre utilization, and the numbers required, all of which need to be presented at a granular level. Hence, it is imperative to broaden the range of metrics incorporated in simulation models to ensure comprehensive resource planning and facilitate effective decision‐making.

An additional crucial consideration pertains to the practical usability of simulation‐based models such as DES, SD, and ABS. These models, due to their inherent complexity, can pose significant challenges for end‐users when attempting to use them in real‐world scenarios. Understanding the intricacies of the simulation's underlying engine is typically limited to developers or experts, making it exceedingly difficult for decision‐makers to effectively utilise these models without a user‐friendly interface. Consequently, simulation‐based models are primarily developed for research purposes rather than being specifically designed to cater to the needs of end‐users. To bridge this gap, efforts must be made to enhance the accessibility and usability of these models, ensuring that decision‐makers can easily comprehend and apply them in practical settings.

Consequently, the main aim of this research is to develop a user‐friendly DST utilising DES. This tool will empower key decision‐makers within the hospital setting to comprehensively evaluate the impact of various interventions on daily operations. It will provide an extensive range of key indicators, encompassing crucial metrics such as required bed capacity, utilization rates of theatres and clinics, staff utilization rates, bed occupancy rates, counts of diagnostic and treatment procedures, revenues, and other relevant measures. By utilising this DST, senior management will gain the necessary support to address bottlenecks prevalent in hospital environments. Moreover, it will expedite the decision‐making process and ultimately enhance outcomes for both patients and staff members. The development of such an intuitive and practical tool holds significant potential for driving improvements in hospital management and overall operational efficiency.

## 3 Methods

### 3.1 The Decision Support Tool

A simulation‐based DST was developed to enhance hospital planning, specifically for use by a significant NHS trust in England, but it can easily be adapted to any setting globally. Its purpose is to promote change and transformation that benefits both the hospital and its patients, especially during times of crisis when staffing shortages, record‐high waiting lists, ongoing strikes, and post‐COVID recovery efforts pose significant challenges.

**FIGURE 1:**
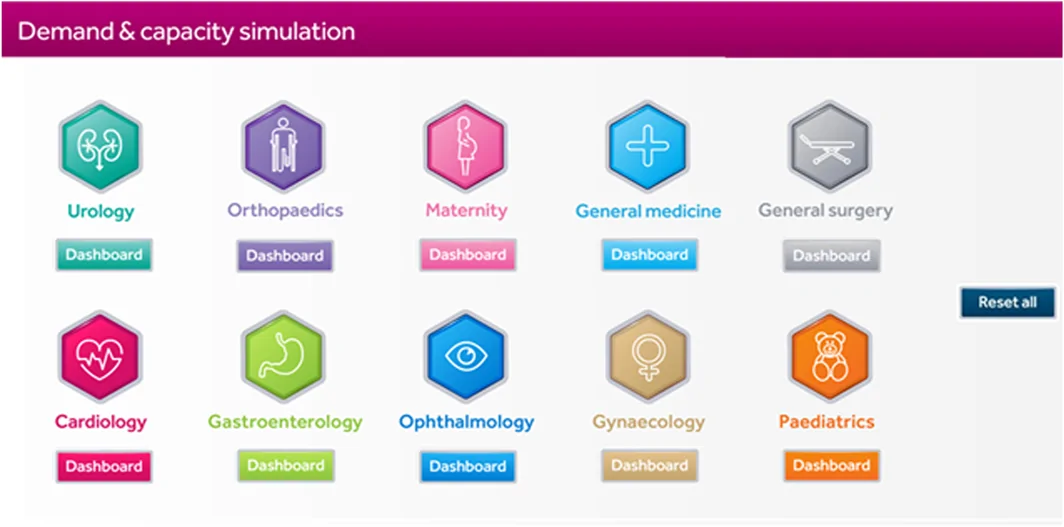
The main DST dashboard.

**FIGURE 2:**
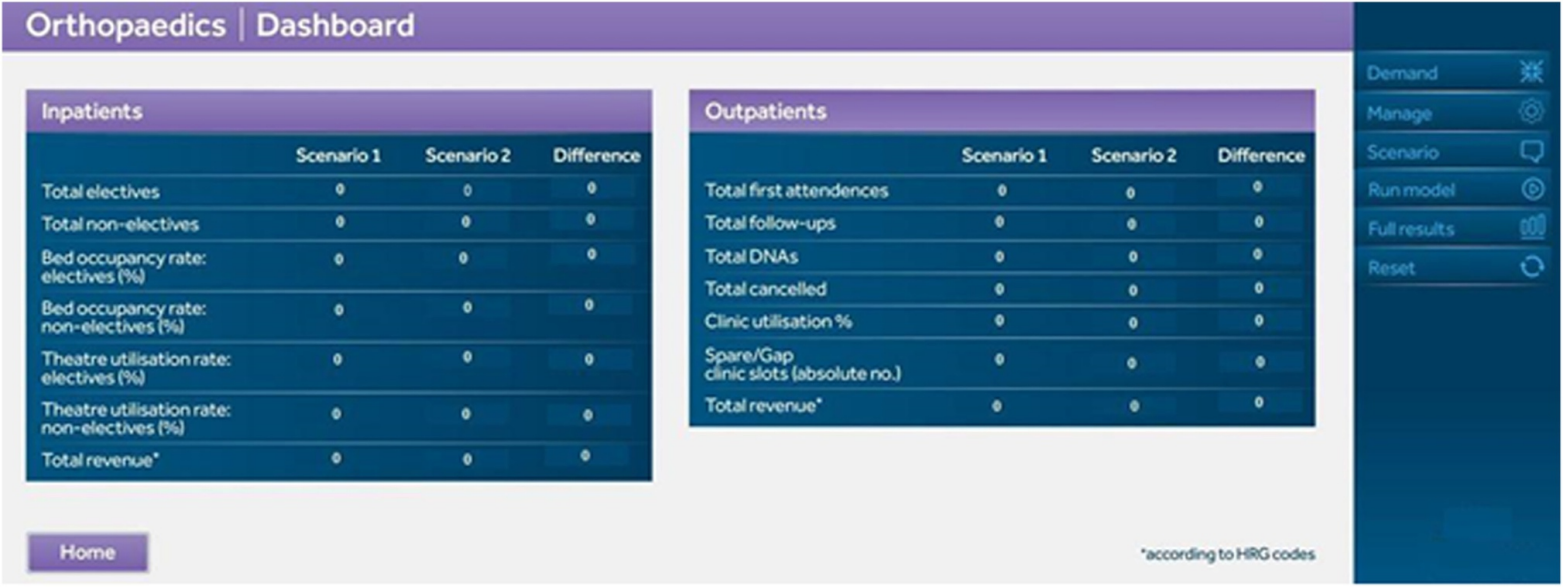
Trauma and Orthopaedics Specialty Dashboard as an example.

To enable non‐experts to run simulations without relying on the research team for future exploration, a graphical user interface was developed and integrated from the outset. This was done because the intended end users (e.g., service managers, doctors) lacked expertise in simulation. See Figures 1 and 2, the main dashboards for the DST and Trauma and Orthopaedics service dashboards, as examples at a specialty level, respectively. The main dashboard of the DST in Figure 1 provides users with a versatile platform to explore individual specialties with ease. Through this intuitive interface, users can effortlessly navigate through different specialty areas, unlocking the ability to conduct simulations and generate essential outputs tailored to their specific needs. This functionality not only allows for a focused examination at the specialty level but also provides a unique opportunity to gain valuable insights into the overall performance of the entire hospital.

Simultaneously, the DST capabilities extend beyond individual specialties, offering a broader perspective on the hospital as a whole. Users can assess the interplay and dependencies between different specialty areas, identifying potential bottlenecks or areas of synergy. This holistic view allows for a comprehensive evaluation of the hospital's performance, uncovering opportunities for optimization and efficiency across multiple departments.

The goal was to design a user‐friendly and functional front‐end interface and simulation controls where users could modify input settings using an Excel spreadsheet. The model can be easily tailored to meet the requirements of entire hospital services, as users have the flexibility to personalise input values for different parameters such as activity‐related inputs, care pathways, healthcare workers, and human and non‐human resources. This customisation ensures the model's adaptability to any hospital setting and makes it highly versatile. A series of workshops were presented by the developer as a training session for key decision‐makers.

The DST offers two configurations for the patient pathway in a hospital setting: scenario 1, which could reflect existing services (e.g., current demand), and scenario 2, a situation in which an intervention is applied, such as an x% increase in non‐elective admissions or the implementation of virtual wards. After the simulation runs for a period of time, the DST generates summary output metrics such as the average bed occupancy rates and theatre and clinic utilization over the simulation period. An extensive range of outputs, including numerical and graphical outputs, is also accessible in the form of an Excel file. For example, the DST estimates the required number of beds and theatre sessions to cope with the demand for all the specialties provided within the hospital (e.g., cardiology, urology, general medicine), as well as clinic utilization in outpatient services and the number of procedures performed within the specialty for each year separately (e.g., 2023–2027). A detailed report on inpatient and outpatient financials for each year is also provided.

### 3.2 Description of the Discrete Event Simulation Model

**FIGURE 3:**
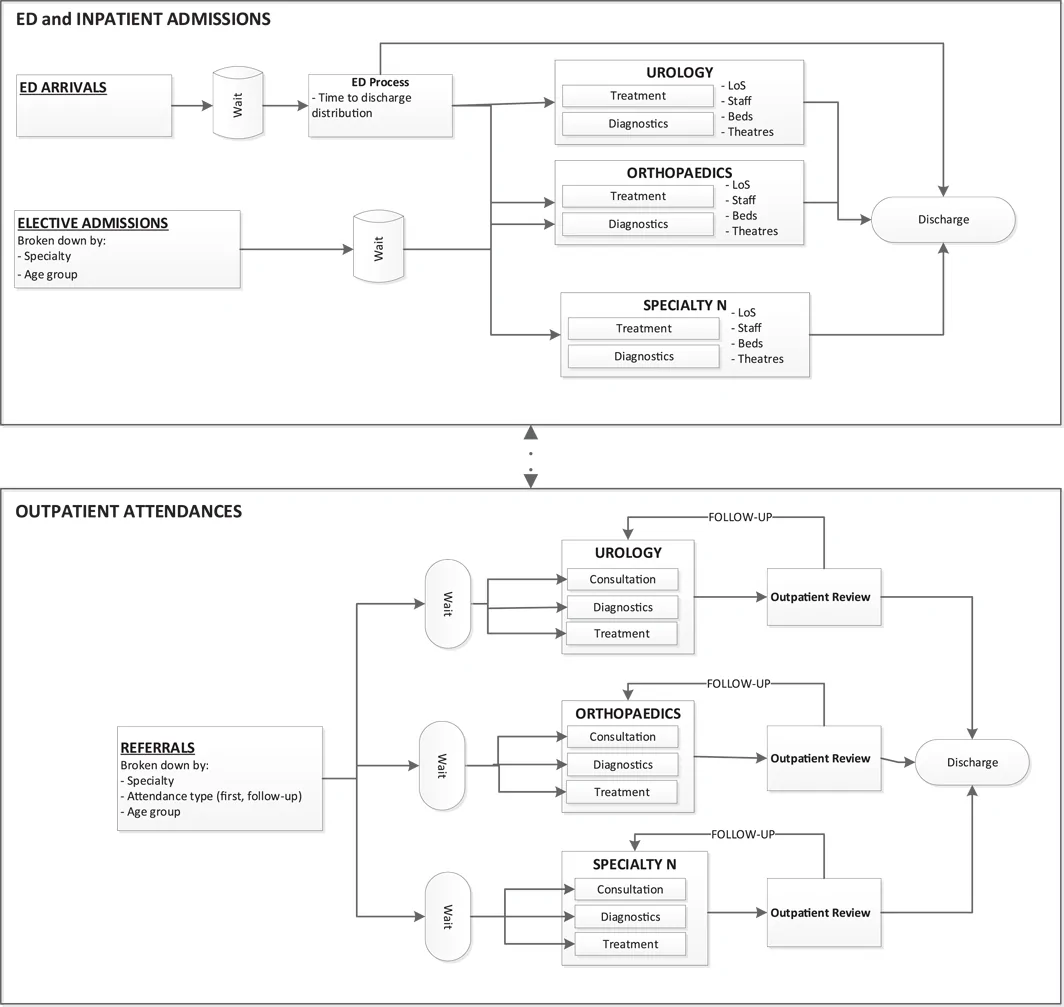
Conceptualised high‐level patient flow.

The participative modelling (PM) technique is used to involve important stakeholders in the modelling process, which also aided in the collection of relevant information for the model's development. Stakeholders were allowed to share their views on the present patient pathway in outpatient service, inpatient care, and the emergency department and evaluate prospective scenarios for change in a supportive atmosphere using the PM approach. Finally, a workshop was arranged to show the updated model to participants and summarise the research. Figure 3 presents a high‐level flow diagram of the patients in the National Health Service (NHS) in England established using the PM approach. There are three primary routes to accessing hospital care, which include emergency department (ED) visits, inpatient admissions, and outpatient attendances. The ED arrival modes consist of walk‐ins, referrals from NHS 111, ambulance handovers, referrals from primary care doctors (such as general practitioners), and community care. Upon registration, patients are triaged by a nurse to determine the urgency of their care needs and are treated based on the availability of necessary resources, such as a doctor and a bed. Once treatment is completed, patients are either discharged back to their usual residence or admitted as non‐elective patients to the relevant specialty. The assignment of necessary resources such as a bed, doctors, nurses, operating theatre (if required), cost of care, diagnostic and treatment procedures, and length of stay (based on a statistical distribution) is carried out during the patient's admission and transfer to the specialty. This process is repeated for all the specialties provided at the hospital.

In addition to non‐elective admissions, patients are admitted to inpatient care through elective admissions, which are planned in advance. An elective admission is initiated either by a consultant‐led referral or by a general practitioner. Following the referral, the patient will wait for a period of time before receiving an appointment date. Once admitted for treatment, necessary resources are assigned to the patient, as in the case of non‐elective admissions.

Outpatient attendances are scheduled in advance for patients to see a consultant or specialist for review, investigation, or treatment. Outpatient attendance can include various services such as consultations, diagnostic tests, therapy sessions, and minor surgical procedures. These appointments are categorised into two types: first attendance, where the patient is referred to be seen by a consultant or specialist for the first time, and follow‐up attendance, which is routinely scheduled to monitor the patient's progress, review test results, adjust treatment plans or medications, or provide further support and advice.

It's important to note that, unlike inpatient admissions (either non‐elective or elective), outpatient attendances do not require an overnight stay, so no bed is assigned. The model used to schedule outpatient appointments takes into account appointment cancellations by either the patient or the hospital and did not attends (DNA) when a patient does not turn up for an appointment. Transitions can also be made between inpatient care and outpatient services.

**FIGURE 4:**
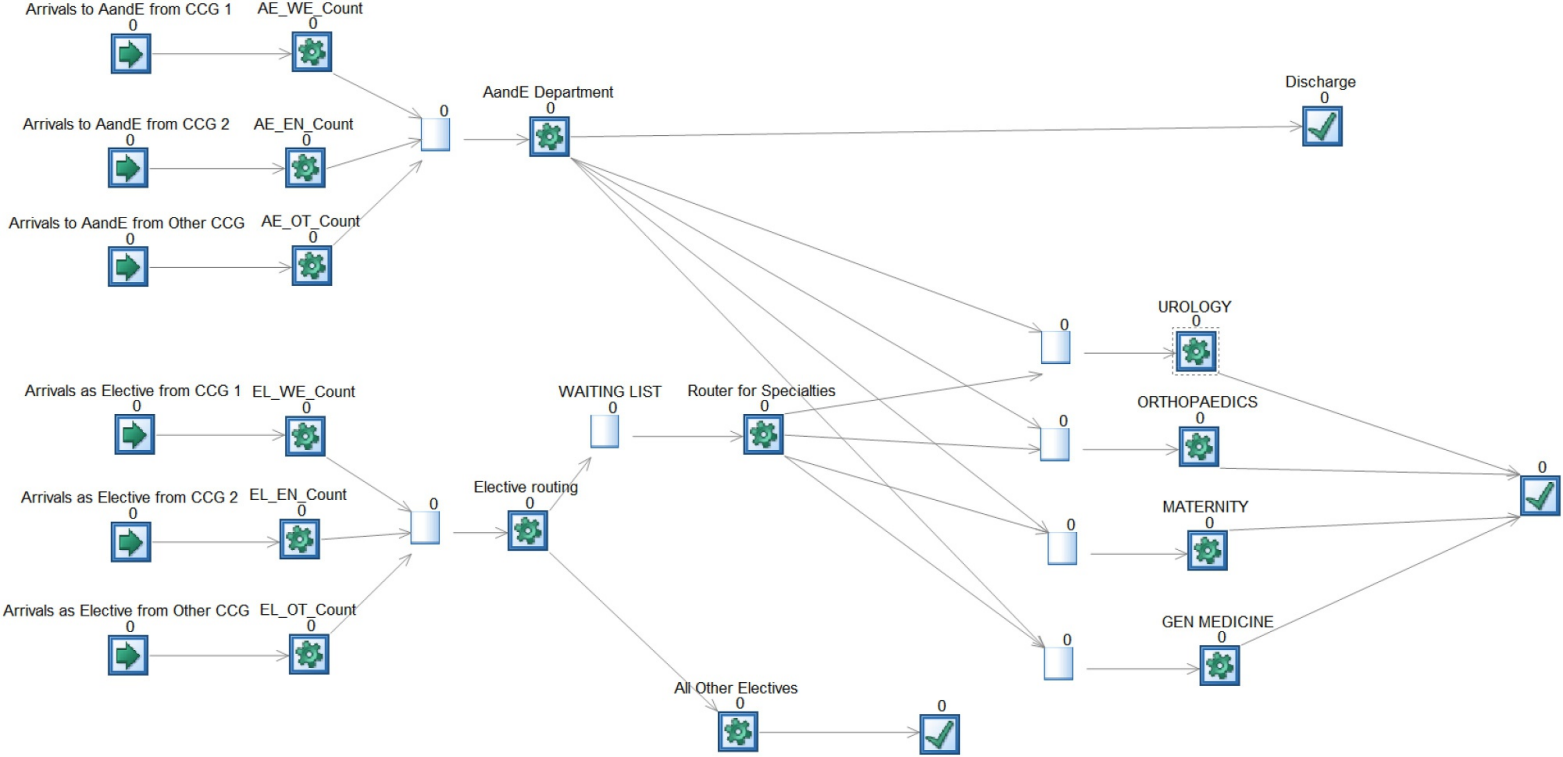
Simul8 screenshot of a small segment of the model for inpatient services.

This process is captured and modelled using the simulation software Simul8 [34], as shown in Figure 4 for all the specialties within the hospital. Individual movements of patients are captured depending on type of admission (elective, non‐elective, first attendance, follow‐up); specialty type (trauma and orthopaedics, cardiology, urology, obstetrics and midwifery, general medicine, general surgery, gynaecology, gastroenterology, ophthalmology, and paediatrics); age group; sex; length of stay, and all the resources required during their stay and treatment (bed, theatre, outpatient clinic, healthcare workers, and cost).

In Figure 4, the Simul8 back‐end engine is showcased, highlighting a specific segment of the extensive Simul8 model. This depiction specifically emphasises inpatient pathways. The diagram provides a simplified, high‐level overview of the processes involved, offering a holistic view of the system. It is important to note that while the figure captures the essence of the processes, it does not represent the full complexity of each specialty's operations. For example, Simul8 portrays the ED as a central hub for emergency cases, emphasising its crucial role. However, in reality, the ED department encompasses various intricate processes such as triage, doctors' consultations, treatments, and diagnostic tests. These complexities are not explicitly captured in the simplified diagram but should be acknowledged when interpreting the model.

Similarly, the inpatient services for each specialty are portrayed as individual processes, resembling simple input‐output systems, where necessary resources such as waiting time, length of stay, time to treatment, bed and theatre requirements, staff, and costing are assigned as necessary. However, in reality, these specialties consist of multifaceted workflows and interactions. While the Simul8 model provides an overview, it does not encompass the intricate details and variations present in actual inpatient department operations.

The implementation of outpatient services is comparable to inpatient services, highlighting their resemblance in terms of simplified representation. Although the Simul8 engine presents an approximation of the outpatient clinic processes, it does not fully capture the diverse range of activities and complexities involved.

Therefore, while Figure 4 offers a valuable high‐level perspective and holistic understanding of the selected specialties and ED, inpatient, and outpatient pathways, it is important to recognise that the Simul8 model simplifies and conceptualises processes to facilitate a broad overview rather than accounting for the full intricacies of each department's operations.

When developing a model for a messy, complex system like a hospital, it is crucial for modelers to aim for a harmonious blend of inclusion and exclusion. Increasing the level of detail in the model leads to a greater number of inputs and requires the collection of relevant data, which could potentially result in slower execution of the model. In our specific situation, we meticulously chose the appropriate degree of granulation that aligned with our objective of evaluating high‐level resource utilization.

### 3.3 Data Sources and Input Parameters

The hospital provided all the data except for cost‐related variables. For each specialty, there are 36 input parameters derived from various categories, including demand, staff, bed and clinic capacity, revenue, and annual operating theatre session capacity, where one or more patients can be operated on within each session. Each category of data is further broken down by inpatient admission level (electives and non‐electives) and outpatient attendance (first and follow‐ups). With 10 specialties, a total of 360 input parameters are estimated (10 x 36), including statistical distributions to capture variation and uncertainty. An example of this is the average length of stay (LoS) of a non‐elective admission (in days) for trauma and orthopaedics (T&O) being 6 days, and the gamma distribution being the best fit for the distribution of LoS based on the data. The hospital's total revenue is closely tied to the Healthcare Resource Groups (HRG) tariffs, as they directly impact its financial earnings. Each patient in the dataset has an HRG code linked with the 2022–23 National Tariff Payment System [35], which reflects the cost of care in GBP. As an illustration, the input parameters associated with the T&O specialty are provided in Table 2. The same is derived and established for all 10 specialties.

**TABLE 2:** The input parameters associated with the Trauma and Orthopaedics specialty.

Parameter	Estimate	Distribution	Resource
Demand			
Activity for financial year 2022/23			
Elective	2740	Fixed	LD
Non elective	1302	Fixed	LD
Outpatient attendances	31,498	Fixed	LD
Outpatient DNAs	1550	Fixed	LD
Outpatient cancellations	4573	Fixed	LD
Elective	2740	Fixed	LD
Expected growth in demand for each of the activities above.	Forecasted based on historical data	Fixed	LD
Staff			
Number of consultants	55.72	Fixed	LD
Number of other doctors	140.55	Fixed	LD
Number of nurses	37.88	Fixed	LD
Number of other support staff	7.05	Fixed	LD
% Of consultants in outpatients	30.65	Fixed	LD
% Of other doctors in outpatients	30.65	Fixed	LD
% Of nurses in outpatients	11.77	Fixed	LD
% Of other support staff in outpatients	100	Fixed	LD
Required nurse to bed ratio	0.8	Fixed	LD
Bed and clinic capacity			
Number of beds available for elective admissions	31	Fixed	LD
Number of beds available for non‐elective admissions	28	Fixed	LD
Total available outpatient clinic slots (per year)	36,700	Fixed	LD
Target bed occupancy	0.85	Fixed	LD
Length of stay and waiting time			
Elective: Mean LoS (in days)	1.75	Gamma distribution	LD
Non‐elective: Mean LoS (in days)	6	Gamma distribution	LD
Average waiting time for an elective admission (in days)	263	Log normal distribution	LD
Average waiting time for first outpatient appointment (in days)	99	Log normal distribution	LD
Average number of follow‐ups	1.8	Poisson	LD
Theatre utilization			
Electives: Theatre capacity expressed in sessions per year	1924	Fixed	LD
Non‐electives: Theatre capacity expressed in sessions per year	1298	Fixed	LD
Electives: Mean number of theatre procedures per session	4.17	Average	LD
Non‐electives: Mean number of theatre procedures per session	2.32	Average	LD
Electives: Proportion of admissions undergo surgical procedure	93	Bernoulli	LD
Non‐electives: Proportion of admissions undergo surgical procedure	90	Bernoulli	LD
Revenue			
Elective tariff (HRG)	£3562.9	Average	NHS england*
Non‐elective tariff (HRG)	£3877.5	Average	NHS england*
First attendance outpatient tariff	£176	Fixed	NHS england*
Follow‐up attendance outpatient tariff	£77	Fixed	NHS england*
Average cost of diagnostic procedures	£29	Average	NHS england*
Average cost of treatment procedures	£139	Average	NHS england*

Abbreviations: LoS: Length of Stay, LD: Local Data, LE: HRG: Healthcare Resource Groups, NHS: National Health Service, *: 2022/23 National Tariff Payment System: Annex A‐National tariff workbook (https://www.england.nhs.uk/wp‐content/uploads/2020/11/22‐23NT_Annex‐A‐National‐tariff‐workbook_Apr22.xlsx).

### 3.4 Forecasted Activity

The extraction process captured the demand for emergency, outpatient, and inpatient services during the financial years 2018–2023. Monthly non‐elective and elective inpatient admissions, ED visits, outpatient attendances, did not attends (DNAs), and cancellations were extracted for each specialty and age category (0–18, 19–64, 65–84, and 85+). For each of the four age groups, as well as outpatient attendances, DNAs, cancellations, and non‐elective and elective admissions (a total of 20 models per specialty), a forecasting model was developed. The forecasted activity between 2023–24 and 2027–28 was estimated by adjusting the predictions for population growth rates (a 5‐year forecast). It should be noted that these age groupings were decided during the PM phase. All statistical distributions and demand forecasts were determined using the statistical software *R* [36].

The selection of models is determined using Akaike's information criterion and the Bayesian information criterion, as well as the forecast accuracy of the model, for example, root mean squared error and mean absolute percentage error. When comparing our projected activity for the 2022–23 period against real‐world activity, our estimates consistently achieved an impressive range of 90%–99%.

### 3.5 Validation and Verification

The hospital simulation model was validated to verify its suitability for further testing in various situations. The model's outputs, such as the number of required beds and theatre sessions, were validated by comparing them to actual data. To do this, the Black‐Box Validation [37] method of validation is utilised, which is a method acknowledged in the field as an effective means of validation.

The findings indicated that the simulation outputs closely matched the observed data, with a difference of less than 5%. This suggests that the model is valid and suitable for scenario runs. As with other simulation models, the rigorous validation process proved the model's precision and dependability for actual applications.

To enhance the robustness of our validation process, white‐box validation [37] is used, which involved meticulously testing every component of the model. This comprehensive approach, implemented during and after development, involved conducting detailed assessments at the individual level. For instance, when evaluating the required number of beds, the model was executed to ensure it generated the expected bed count.

Furthermore, we recognized the significance of achieving face validity as an additional technique to validate simulation models. The model's reasonableness and alignment with real‐world acute care systems were evaluated through individual consultations with the hospital staff, followed by a workshop involving the entire group. This inclusive approach led to unanimous agreement among NHS staff during the stakeholder engagement meetings, confirming that the model's structure was highly representative of the actual operational system.

During the course of the study, the continued involvement of key stakeholders greatly enhanced the validity of the model, strengthening its dependability and credibility.

## 4 Results

The generic hospital DST holds immense value as it enables the evaluation of diverse changes in input parameters and their potential influence on numerous model outputs. The DST has a large number of input parameters, which means that it can be challenging to identify and analyse the specific factors that may affect the hospital's operations in the future.

To address this challenge, some of the input parameters were systematically altered to evaluate the potential changes in the hospital's operations between 2023 and 2027. These changes were focused on exploring scenarios related to the provision of resources necessary to alleviate the pressures on the system, such as the growing waiting list, shortage of staff, and rising demand for services.

By analysing the impact of these scenarios on the model outputs, we can better understand how the hospital may need to adapt in the coming years to meet the demands of patients and the wider healthcare system. This information can be utilised to steer decision‐making procedures and ensure the hospital's readiness to address the changing requirements of its patients and the broader community.

To better understand the potential effects that various scenarios may have on the hospital's operations from 2023 to 2027, we have formulated four scenarios in collaboration with the hospital's management.

1. **Baseline Scenario**: This scenario involves maintaining the hospital as is, with no changes to the current operating procedures and patient flow.
2. **Non‐elective Admissions Increase**: In this scenario, there would be an average of 2.5% increase in non‐elective admissions across all specialties, which is in line with the forecasts.
3. **Elective Admissions and Outpatient Attendances Increase**: This scenario involves a 10% increase in inpatient elective admissions and outpatient attendances for the first two years (2023–2024). The aim is to address the backlog of patients on the waiting list and gradually decrease admissions and attendances over the next three years (2025–2027) with the goal of achieving the 18‐week target set by NHS England.
4. **Elective Admissions and Outpatient Attendances Increase with Virtual Wards**: In this scenario, scenarios 2 and 3 were combined to tackle the exceedingly high number of patients waiting for treatment as elective admissions and outpatient attendances. Once the waiting list has been reduced, we introduce virtual wards to further decrease outpatient attendances by 15% across all specialties over the next three years (2025–2027). Note that virtual wards are currently undergoing a trial period at the hospital. Therefore, it is expected that full implementation of virtual wards may take until the year 2025.

In the NHS, a virtual ward is a care model that uses telemedicine technologies to provide support and monitoring to patients in their own homes rather than requiring them to stay in a hospital bed. It is a method of extending hospital treatment into the community and can be used for patients recovering from sickness or surgery, as well as people with complicated requirements who require continuous medical care. A multidisciplinary team of healthcare experts, including doctors, nurses, and therapists, often manages the virtual ward. They remotely monitor the patient's status and provide essential therapies as needed. Evidence suggests that virtual wards can assist to reduce hospital admissions, enhance patient outcomes, and increase the efficiency of healthcare services by delivering treatment in the patient's home [38].

By examining the scenarios listed above, where the introduction of virtual wards is considered in scenario 4, we can evaluate the potential impact of changes to the hospital's operations on many key performance metrics, such as the waiting list, resource utilization, and outcomes. We will focus on the required bed capacity, outpatient clinic utilization rates (in percentages), theatre session requirements, and activity. This information will help the management make informed decisions about the hospital's future operations and prepare for the challenges that lie ahead.

Our experimental design involved creating scenarios cumulatively, meaning that each scenario is built upon the changes introduced in the preceding scenario. For example, the adjustments made in scenario three included those made in scenario 2. Consequently, the modifications introduced in Scenario four encompassed all the changes made in the previous scenarios. This methodology allowed us to identify which scenarios produced the most significant effects by building upon each other in a progressive manner.

As a significant amount of data is generated, the outputs for one of the chosen specialties is presented, namely T&O. However, the same range of results, and even more, are generated for the other nine specialties. In addition to this, hospital‐level outputs are displayed to provide an overview of the impact of the scenarios on resources across the entire hospital, rather than solely on a single specialty. This will enable us to gain a broader perspective on the overall impact of the scenarios.

**FIGURE 5:**
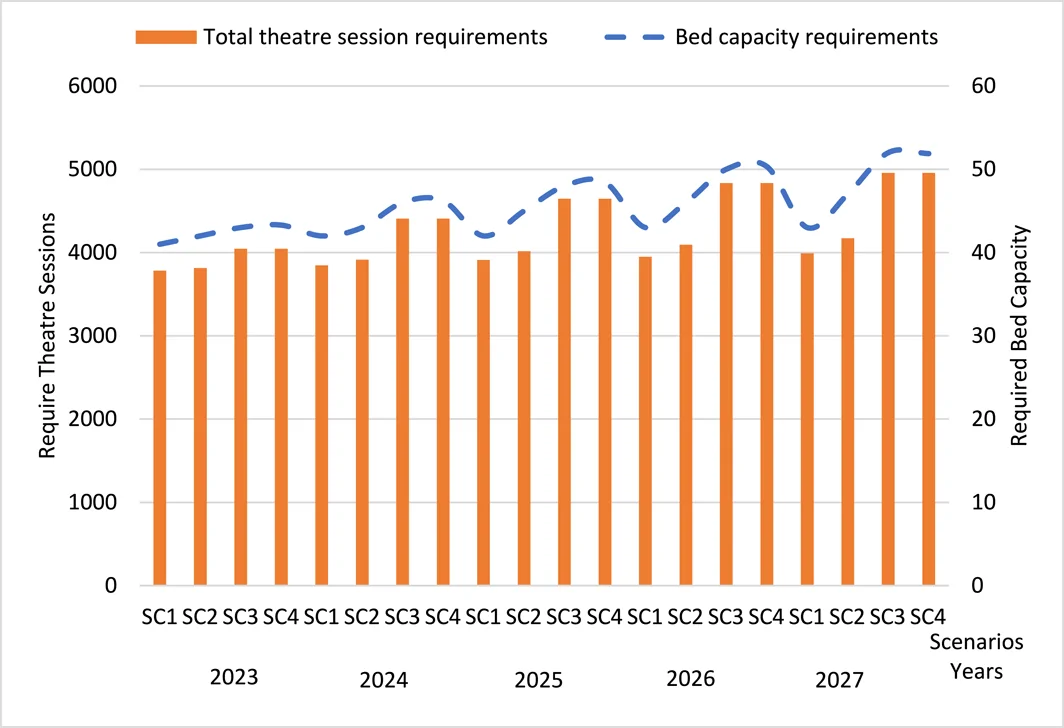
T&O theatre session and bed capacity requirements for the period 2023–2027 for each scenario. SC1–SC4 are scenarios 1–4.

**FIGURE 6:**
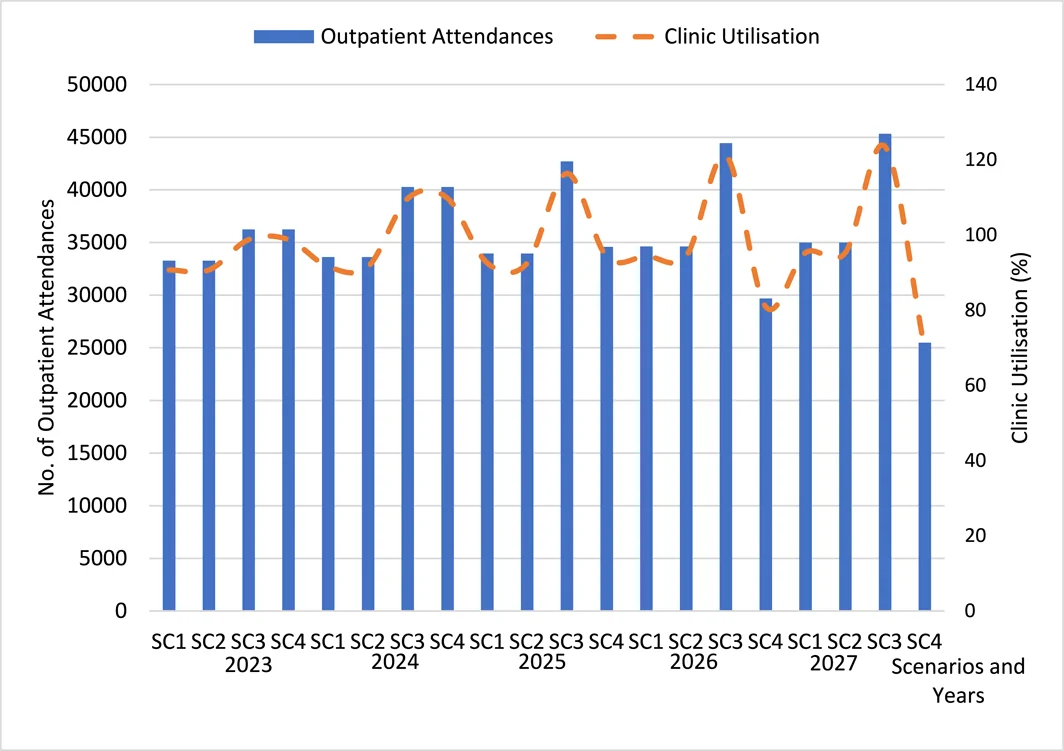
The expected number of T&O outpatient attendances (first and follow‐ups) and clinic utilization (%) for the period 2023–2027 for each scenario. SC1–SC4 are scenarios 1–4.

In accordance with scenario 2 (SC2), a year‐on‐year 2.5% rise in non‐elective admissions for T&O leads to an increase in bed requirements from 42 to 47 (12%) over the five‐year span (see Figure 5). Additionally, theatre session requirements increase from 3814 (current year) to 4173 sessions (9.4%) by 2027. See Figure 5 for details. The T&O department anticipates approximately 4145 inpatient admissions in 2023, which are projected to rise to 4538 by 2027. It is expected that there will be a yearly increase of 1% in clinic utilization, rising from the current 91%–95% by 2027 (see Figure 6).

Scenario 3 is a combination of Scenario 2 with a yearly 10% increase in inpatient elective admissions and outpatient attendances. This scenario has a significant impact on the T&O department, resulting in an increase in demand from 4394 inpatient admissions in 2023 to 5379 by 2027, which is a 22.4% increase. As a result, the required bed capacity increased from 43 to 52 (21% increase) over the five‐year period (Figure 5). However, the implementation of this scenario leads to maximum clinic utilization (99%), with no room for any spare capacity beyond 2023. This situation exacerbates clinic utilization well above capacity, reaching 110% in 2024 and 124% by 2027 (Figure 6). Consequently, the hospital will require additional clinic slots to meet this level of demand and alleviate the pressure on the waiting list. The gradual decrease in outpatient attendances from years 3–5 (2025–2027) has some impact on the reduction of clinic utilization.

The implementation of virtual wards on T&O in Scenario 4 has a considerable effect on the utilization of clinics, leading to a significant decrease in the third, fourth, and fifth years. In 2023, the clinic utilization rate of the hospital will be 99%. However, it shows a rise to 110% in 2024 before declining to 69% in 2027.

Table 3 presents a comprehensive breakdown of the expected level of hospital activity for the upcoming period of 2023–2027. The table encompasses resource‐related metrics and accounts for 10 specialties. Currently (SC1), the hospital is operating at an excessively high capacity of 105% clinic utilization, surpassing the recommended level. It is projected that by the end of year 5, this figure will further escalate to 117%, indicating a critical situation. Furthermore, the implementation of SC2 and SC3 is likely to worsen the current scenario, leading to an anticipated rise in clinic utilization of up to 142% by 2027. However, if SC4 is implemented, the hospital can expect some relief by 2026, with a projected decrease in clinic utilization down to 95%.

**TABLE 3:** The number of inpatient admissions (electives and non‐electives), outpatient attendances (first attendances and follow‐ups), required bed capacity, outpatient clinic slot utilization (%) and the total theatre session requirements for each scenario for the period 2023–2027.

Year	Scenarios	Inpatient admissions	Outpatient attendances	Bed capacity requirements	Clinic utilization (%)	Total theatre session requirements
2023	SC1	68,439	214,749	534	105	15,005
	SC2	69,430	215,705	546	106	15,126
	SC3	73,047	231,978	561	114	16,100
	SC4	72,317	229,658	555	113	15,939
2024	SC1	69,238	219,951	540	108	15,280
	SC2	70,347	217,752	552	109	15,537
	SC3	78,049	257,286	584	127	17,558
	SC4	78,733	259,288	589	128	17,712
2025	SC1	70,018	225,133	546	110	15,541
	SC2	72,142	226,631	571	111	15,930
	SC3	82,159	272,611	612	134	18,515
	SC4	81,611	221,442	608	109	18,392
2026	SC1	70,806	230,711	552	114	15,788
	SC2	73,098	230,480	577	113	16,330
	SC3	84,573	283,429	625	140	19,252
	SC4	83,748	190,671	619	93	19,064
2027	SC1	71,586	236,546	558	117	16,034
	SC2	74,966	238,231	596	118	16,728
	SC3	86,936	287,506	646	142	19,734
	SC4	86,359	171,498	642	80	19,603

It is important to note that all scenarios will experience a substantial increase in bed capacity and theatre session requirements by the end of year 5. This upsurge is expected to continue until the end of the simulation period.

**FIGURE 7:**
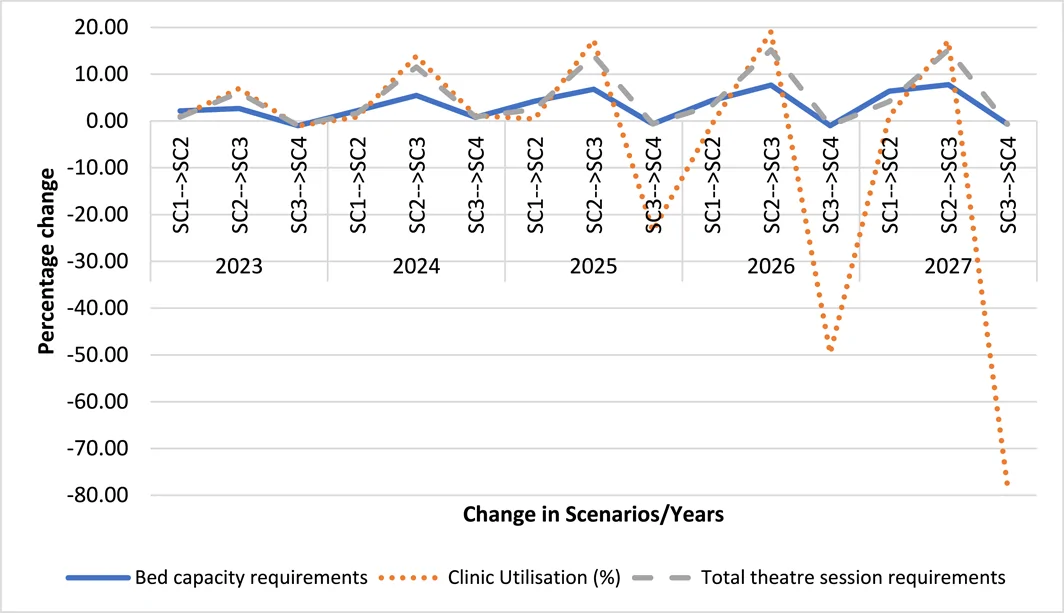
Percentage change in the required bed capacity, clinic utilisation and theatre session utilization between the scenarios for the years 2023–2027. SC1 – SC4 are Scenarios 1‐ 4.

Figure 7 depicts the projected percentage change across different scenarios. It is noteworthy that the implementation of virtual wards comes at a cost to the hospital, but it is also the most effective intervention to improve hospital capacity. For instance, when comparing SC3 to SC4 in the years 2025, 2026, and 2027, the expected percentage change is −23.5%, −50%, and −78%, respectively. The negative sign indicates a reduction in clinic utilization from the previous year, despite the predicted increase in bed capacity and theatre session requirements. This suggests that virtual wards are a promising solution to enhance the efficiency of the hospital while simultaneously improving patient outcomes.

## 5 Discussion

This study's DST addresses a major topic in healthcare management, specifically policies linked to service redesign and their influence on crucial metrics concerning the demand and capacity of healthcare services. The urgent need for an easy‐to‐use DST by decision makers is especially clear in the current environment of healthcare delivery, which is undergoing problems due to increased demand for health services combined with the detrimental impact of the COVID‐19 pandemic. These crises are exacerbated by variables such as tighter public finances as a result of economic downturns, higher living expenses, and a scarcity of healthcare employees. Efforts should be made to enhance the efficiency and effectiveness of healthcare service provision and delivery. The DST provides a timely solution for key opinion leaders to improve the challenges experienced by staff and patients within the healthcare industry because it provides vital information into how a healthcare system operates in relation to key metrics, such as utilization of resources, revenue generation, and diagnostic and treatment procedures. Senior decision‐makers can use this tool to restructure their services in an evidence‐based manner, seeking to provide the best possible care with the utmost efficiency and effectiveness.

The model integrates both resource‐related inputs (e.g., beds, staff, operating theatres, diagnostics, clinics) and patient‐related inputs (e.g., demographics, patient severity, comorbidities, waiting times, length of stay, socioeconomic status). By incorporating these inputs, our simulation is capable of generating a range of outputs, including patient outcomes (e.g., mortality rates, readmissions, disease progression, quality‐adjusted life years [QALYs]) and resource utilisation metrics. While the primary focus of this article has been on operational metrics to support senior management in streamlining hospital planning processes, it is important to acknowledge that the simulation also robustly generates patient outcome metrics. These outputs are equally significant and provide a comprehensive understanding of both the operational efficiency and patient care quality within the hospital. Future research could explore the patient outcome metrics in greater detail, highlighting the model's potential to contribute to both operational and clinical decision‐making.

Potential solutions are presented in this study to alleviate the strain on acute services. These solutions involve leveraging virtual wards to enhance the quality, efficiency, and productivity of elective admissions and outpatient attendances. Virtual wards have been successfully implemented by hospitals in the UK as a long‐term management strategy, demonstrating their safety and effectiveness (e.g., increased patient satisfaction) when implemented correctly [39]. However, the impact of virtual wards on services has not been previously studied.

Spreadsheet‐based models are extensively utilised within the NHS for decision‐making purposes, often relying heavily on averages such as the mean number of attendances, the mean LoS, and the mean utilization rates of resources. However, relying solely on averages can lead to significantly flawed predictions, commonly referred to as the 'error of averages' [40]. Considering the substantial financial turnover of hundreds of millions of pounds for services like NHS hospitals, it becomes imperative to prioritise accuracy in these models.

In contrast to spreadsheet models, DES possesses a unique strength: its capacity to effectively capture variability. This is particularly valuable when considering complex factors like the length of stay in inpatient services, which plays a crucial role in determining throughput, resource utilization, and financial implications. Merely assuming an average length of stay, such as 1.75 days in T&O, can be highly misleading. By employing DES, we can account for the inherent variability in length of stay and generate more accurate predictions and assessments of the associated outcomes and costs. This allows us to make informed decisions and allocate resources more effectively within the healthcare system.

Therefore, the research study adds to the data supporting the use of simulation approaches, specifically DES, for healthcare decision‐making. DES is a useful tool that may represent the complex structure of patients' pathways within a complex system like a hospital and predict the likely results of interventions even before they are implemented in practice. This aspect of DES provides essential information and evidence, allowing key decision‐makers to make informed decisions at times of urgency and need. Furthermore, DES can reduce the risks involved in the implementation of alternative interventions and avoid costly experimentation and trial‐and‐error procedures that are no longer possible due to healthcare service limits.

One critical and often overlooked aspect that demands greater attention from researchers is the conversion of the intricate back‐end simulation engine into a user‐friendly DST. The primary objective of this DST is to enable end‐users, such as service managers and clinicians, who lack simulation experience to evaluate the potential effects of changes within the patient pathway using a validated simulation model. This specific aspect is a significant deficiency in numerous simulation‐based models, which tend to be predominantly developed for research purposes. However, it is imperative that models of this nature are designed and developed, as outlined in this study, using a participative modelling approach that actively involves the end‐users. By addressing this critical gap, we can enhance the practicality and applicability of simulation models for real‐world decision‐making.

The outputs in the form of graphical outputs and tables are exhaustive, providing all the necessary metrics for the entire hospital that both executives and specialists can understand, thereby accelerating the rate of change in hospitals. A major NHS hospital in England is currently using the DST to assist with the current challenges, primarily revolving around post‐COVID‐19 recovery and the reduction of the waiting list. The DST is a generic solution that can be adapted to other healthcare services, such as emergency departments, inpatient services, and outpatient services, which exhibit similar patterns and characteristics worldwide.

A limitation of the study is that obtaining the hospital's complete datasets on activity and resources is necessary to power the DST, which can sometimes be difficult to obtain. Moreover, the simulation model falls short in capturing the intricate dynamics inherent in emergency departments, encompassing crucial components like triage, consultations, treatment, and diagnostic services. These fundamental processes are universally implemented by emergency departments across the globe, serving as critical pillars of their functioning. By omitting these complexities, the current simulation model fails to encapsulate the multifaceted nature of real‐world emergency departments, thereby limiting its effectiveness in accurately representing the intricate interplay of factors that influence patient flow and overall operational outcomes.

The DST will be expanded in the future to include these complexities as well as individual patient factors that may influence the pathway, namely socioeconomic status and comorbidities. Furthermore, to comprehensively evaluate the cost‐effectiveness of implementing a virtual ward, it remains essential to factor in the associated costs impacting various facets of hospital operations.

## Funding

The author has nothing to report.

## Ethics Statement

The author has nothing to report.

## Conflicts of Interest

The author declares no conflicts of interest.

## Data Availability

Research data are not shared.
